# Supplementary material for: Splice-Junction-Based Mapping of Alternative Isoforms in the Human Proteome
Source: Cell Rep. Author manuscript; Available in PMC 2020 Jan 15. (PMC6961840; doi:10.1016/j.celrep.2019.11.026)
Supplement: 2 [file NIHMS1546469-supplement-2.zip › DF1/PXD010154/Heart/Heart_37_SYNRG_FSTASASHSVPEAGPSLEEK.pdf]

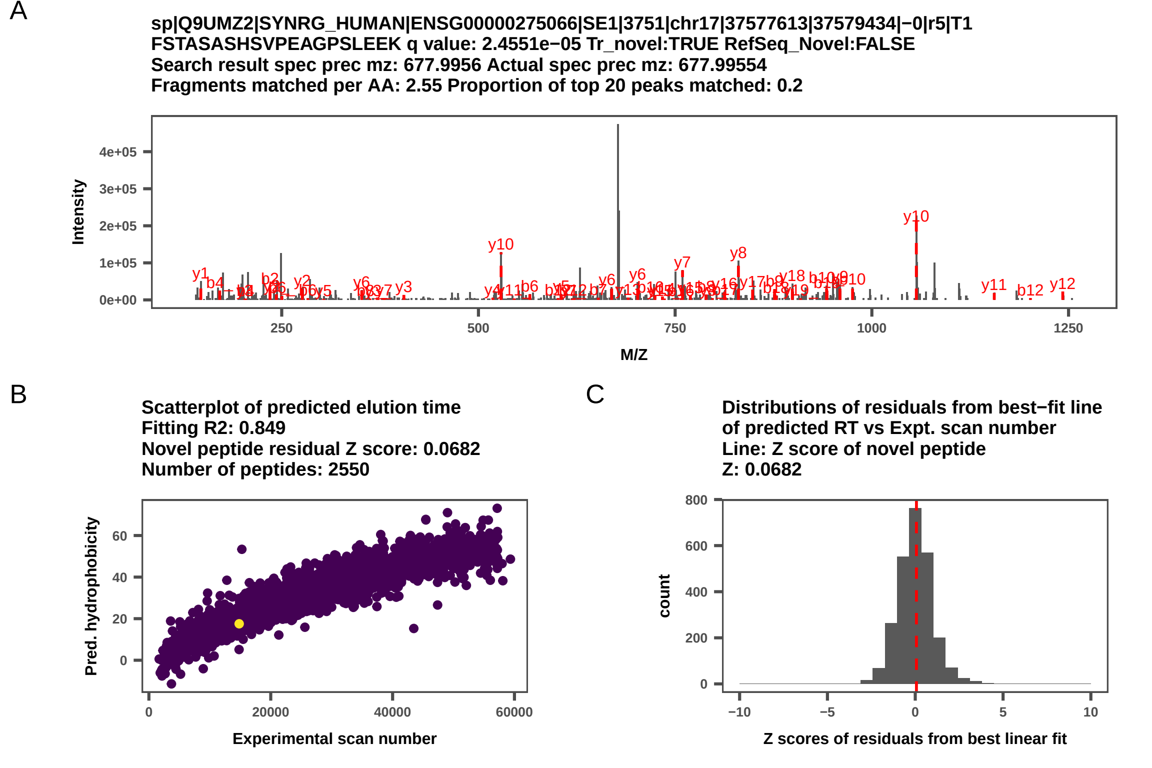
A
sp|Q9UMZ2|SYNRG_HUMAN|ENSG00000275066|SE1|3751|chr17|37577613|37579434|−0|r5|T1
FSTASASHSVPEAGPSLEEK q value: 2.4551e−05 Tr_novel:TRUE RefSeq_Novel:FALSE
Search result spec prec mz: 677.9956 Actual spec prec mz: 677.99554
Fragments matched per AA: 2.55 Proportion of top 20 peaks matched: 0.2
Intensity
4e+05
3e+05
2e+05
1e+05
0e+00
y10
y8
y7
y6
y11
b12
y12
y1
y2
y3
y5
b6
250
500
750
1000
1250
M/Z
B
Scatterplot of predicted elution time
Fitting R2: 0.849
Novel peptide residual Z score: 0.0682
Number of peptides: 2550
Pred. hydrophobicity
60
40
20
0
0
20000
40000
60000
Experimental scan number
C
Distributions of residuals from best−fit line
of predicted RT vs Expt. scan number
Line: Z score of novel peptide
Z: 0.0682
count
800
600
400
200
0
−10
−5
0
5
10
Z scores of residuals from best linear fit
